# Enteric Budesonide in Transplant and Native IgA Nephropathy: Real-World Clinical Practice

**DOI:** 10.3389/ti.2022.10693

**Published:** 2022-10-14

**Authors:** Marina Lopez-Martinez, Irina Torres, Sheila Bermejo, Francesc Moreso, Clara Garcia-Carro, Ander Vergara, Natalia Ramos, Manel Perello, Alejandra Gabaldon, M. Antonieta Azancot, Monica Bolufer, Nestor Toapanta, Oriol Bestard, Irene Agraz-Pamplona, Maria Jose Soler

**Affiliations:** ^1^ Department of Nephrology, Vall d’Hebron University Hospital, Barcelona, Spain; ^2^ Centro de Referencia en Enfermedad Glomerular Compleja del Sistema Nacional de Salud (CSUR), Vall d’Hebron University Hospital, Barcelona, Spain; ^3^ Department of Nephrology, San Carlos Clinical University Hospital, Madrid, Spain; ^4^ Centro de Referencia en Enfermedad Glomerular Compleja del Sistema Nacional de Salud (CSUR), San Carlos Clinical University Hospital, Madrid, Spain; ^5^ Department of Pathology, Vall d’Hebron University Hospital, Barcelona, Spain

**Keywords:** IgA nephropathy, IgAN recurrence in transplant kidney, proteinuria reduction, TRF-budesonide, CKD progression

Dear Editors,

Targeted-release-formulation of budesonide (TRF-budesonide) has demonstrated promising results in terms of proteinuria and renal function in patients with immunoglobulin A nephropathy (IgAN) (1). Regarding its well tolerance, enteric budesonide may become the first step of immunosuppressive treatment of IgAN, although real world clinical practice publications are lacking (2-4). We evaluated the effect of budesonide in our cohort of patients affected by IgAN. We included all patients, either transplanted kidney or native, which were diagnosed of IgAN and were treated with enteric budesonide in our center from December 2017 to January 2022. At baseline clinical and analytical parameters were collected during the next 3, 6, 12, and 24 months. We also assessed the occurrence of budesonide-related adverse events.

A total of 14 patients were included in the study. Nine of the patients had IgAN in their native kidneys (7 males) and 5 were transplanted (5 males), age of 46 ± 17.21 years. The relative decrease of proteinuria was of 33.1% and 54.6% after 3 and 6 months of treatment with budesonide, respectively (*p* < 0.05) (Table 1). Evaluating native and transplant kidney separately, proteinuria in transplant kidney also significantly decreased (26.7%) after 3 months of treatment (Table 1). These results are in line with previous literature (2–6). There is increasing evidence about the role of gut-associated lymphoid tissue and complexes with Gd-IgA1 deposition in IgAN pathogenesis (7). The first study that evaluated TRF-budesonide published a significant albuminuria reduction of 40% in 16 patients with IgAN after 2 months of treatment (2). Afterwards, the phase 2b clinical trial NEFIGAN demonstrated significant proteinuria reduction (21%–27%) in 199 patients with IgAN after 9 months of treatment with TRF-budesonide (5). This latest trial justified to carry out the phase 3 trial NEFIGARD, where TRF-budesonide significantly reduced UPCR by 27% of 199 patients (6). There is only another retrospective study that evaluated the effect of TRF-budesonide in native kidneys IgAN with significant proteinuria reduction (3). This constant effect of local budesonide in proteinuria reduction is quite remarkable, as proteinuria is considered as the main sign of disease progression in IgAN (7) and a surrogate marker of kidney outcome in IgAN (8).

**TABLE 1:** Change from baseline of analytical parameters.

	Baseline	3 months	6 months	12 months	24 months
UPCR native-kidney (Relative decrease %)	1.2 (0.70–2.80)	0.81 (0.8–0.93) ‒32.5	0.53 (0.18–0.85) ‒55.8	1.10 (0.16–1.35) ‒8.3	0.74 (0.30–1.70) ‒38.3
UPCR transplant-kidney (Relative decrease %)	1.5 (0.78–4.3)	1.1 (0.39–1.55)* ‒26.7	0.59 (0.17–1.40) ‒60.7	0.75 (0.16–1.33) ‒50.0	1.27 (0.55–1.70) ‒15.3
UPCR total (Relative decrease %)	1.3 (0.72–4.23)	0.87 (0.54–1.18)* ‒33.1	0.59 (0.17–0.85)* ‒54.6	1.10 (0.21–1.30) ‒15.4	0.92 (0.36–1.83) ‒29.2
Creatinine (mg/dl)	1.66 ± 0.83	1.89 ± 1.62	1.41 ± 0.53	1.37 ± 0.60	1.78 ± 0.49
CKD-EPI (ml/min)	57.14 ± 24.49	57.07 ± 27.71	62.33 ± 23.00	65.63 ± 25.39	57.40 ± 25.47
Haematuria (yes %)	9 (69.2)	8 (61.5)	5 (50)	6 (75)	5 (100)
BMI (kg/m^2^)	27.19 ± 6.70	26.25 ± 6.49	28.95 ± 7.68	23.93 ± 2.06	23.3 ± 2.48
LDL (mg/dl)	112.73 ± 50.53	99.18 ± 31.74	91.63 ± 25.89	101.67 ± 39.64	108.40 ± 38.06
HbA1c (%)	5.89 ± 0.98	5.40 ± 0.30	6.07 ± 1.57	5.53 ± 0.85	5.22 ± 0.40
Clinical adverse events (yes %)	0	0	0	0	0

Median values (interquartile range 25/75 within parentheses) and relative decrease percentage of urine protein to creatinine ratio (UPCR), mean values (SD) of creatinine and estimated glomerular filtration rate by Chronic Kidney Disease Epidemiology Collaboration Equation (CKD-EPI), presence of haematuria, body mass index (BMI), low density lipoprotein (LDL), HbA1c, and clinical adverse events before and after 3-, 6-, 12-, and 24-month treatment with budesonide was started. *p < 0.05 versus baseline.

To our knowledge, there is only a case report published that described a successful post-transplant IgAN treated with TRF-budesonide (4). As 58% of IgAN recurs post-transplant (4,9) and 20%–40% progress to end-stage chronic kidney disease (9,10), TRF-budesonide could be a promising effective treatment in these patients. None of the patients experimented any adverse event. HbA1c, LDL and body mass index, whose increment could be considered as adverse events of steroid therapy, remained stable (Table 1). NEFIGAN and NEFIGARD trials corroborate this well tolerance (5,6). Local steroid therapy, like enteric budesonide, provides the immunosuppressive result directly in the IgAN origin and avoids serious side effects usually present in systemic steroid treatment.

Our results support that TRF-budesonide causes significant proteinuria reduction and maintain eGFR stable without adverse events in IgAN. Remarkably, the effect of local steroid treatment in transplant kidneys should also be analyzed in proper designed randomized clinical trials. Targeting intestinal mucosal immune system seems to be a good therapeutic strategy of IgAN treatment which will probably replace systemic steroids.

## Data Availability

The original contributions presented in the study are included in the article/supplementary material, further inquiries can be directed to the corresponding authors.
