# Corrigendum: Enteric Budesonide in Transplant and Native IgA Nephropathy: Real-World Clinical Practice

**DOI:** 10.3389/ti.2022.11073

**Published:** 2023-01-05

**Authors:** Marina Lopez-Martinez, Irina Torres, Sheila Bermejo, Francesc Moreso, Clara Garcia-Carro, Ander Vergara, Natalia Ramos, Manel Perello, Alejandra Gabaldon, M. Antonieta Azancot, Monica Bolufer, Nestor Toapanta, Oriol Bestard, Irene Agraz-Pamplona, Maria Jose Soler

**Affiliations:** ^1^ Department of Nephrology, Vall d’Hebron University Hospital, Barcelona, Spain; ^2^ Centro de Referencia en Enfermedad Glomerular Compleja del Sistema Nacional de Salud (CSUR), Vall d’Hebron University Hospital, Barcelona, Spain; ^3^ Department of Nephrology, San Carlos Clinical University Hospital, Madrid, Spain; ^4^ Centro de Referencia en Enfermedad Glomerular Compleja del Sistema Nacional de Salud (CSUR), San Carlos Clinical University Hospital, Madrid, Spain; ^5^ Department of Pathology, Vall d’Hebron University Hospital, Barcelona, Spain

**Keywords:** IgA nephropathy, IgAN recurrence in transplant kidney, proteinuria reduction, TRF-budesonide, CKD progression

In the original article, there was a mistake in Table 1 as published. Since publication, it has been noticed that the rows “BMI (Kg/m^2^)” and “LDL (mg/dl)” incorrectly contained repeated data. The corrected version of Table 1 is shown below.

**TABLE 1:** Change from baseline of analytical parameters.

	Baseline	3 months	6 months	12 months	24 months
UPCR native-kidney (Relative decrease %)	1.2 (0.70–2.80)	0.81 (0.8–0.93) ‒32.5	0.53 (0.18–0.85) ‒55.8	1.10 (0.16–1.35) ‒8.3	0.74 (0.30–1.70) ‒38.3
UPCR transplant-kidney (Relative decrease %)	1.5 (0.78–4.3)	1.1 (0.39–1.55)* ‒26.7	0.59 (0.17–1.40) ‒60.7	0.75 (0.16–1.33) ‒50.0	1.27 (0.55–1.70) ‒15.3
UPCR total (Relative decrease %)	1.3 (0.72–4.23)	0.87 (0.54–1.18)* ‒33.1	0.59 (0.17–0.85)* ‒54.6	1.10 (0.21–1.30) ‒15.4	0.92 (0.36–1.83) ‒29.2
Creatinine (mg/dl)	1.66 ± 0.83	1.89 ± 1.62	1.41 ± 0.53	1.37 ± 0.60	1.78 ± 0.49
CKD-EPI (ml/min)	57.14 ± 24.49	57.07 ± 27.71	62.33 ± 23.00	65.63 ± 25.39	57.40 ± 25.47
Haematuria (yes %)	9 (69.2)	8 (61.5)	5 (50)	6 (75)	5 (100)
BMI (kg/m^2^)	27.19 ± 6.70	26.25 ± 6.49	28.95 ± 7.68	23.93 ± 2.06	23.3 ± 2.48
LDL (mg/dl)	112.73 ± 50.53	99.18 ± 31.74	91.63 ± 25.89	101.67 ± 39.64	108.40 ± 38.06
HbA1c (%)	5.89 ± 0.98	5.40 ± 0.30	6.07 ± 1.57	5.53 ± 0.85	5.22 ± 0.40
Clinical adverse events (yes %)	0	0	0	0	0

Median values (interquartile range 25/75 within parentheses) and relative decrease percentage of urine protein to creatinine ratio (UPCR), mean values (SD) of creatinine and estimated glomerular filtration rate by Chronic Kidney Disease Epidemiology Collaboration Equation (CKD-EPI), presence of haematuria, body mass index (BMI), low density lipoprotein (LDL), HbA1c, and clinical adverse events before and after 3-, 6-, 12-, and 24-month treatment with budesonide was started. *p < 0.05 versus baseline.

The authors apologize for this error and state that this does not change the scientific conclusions of the article in any way. The original article has been updated.

